# CRISPR/Cas9-based gene-editing platform development by targeting genes encoding magnesium chelatase and phytoene desaturase in sugarbeet (*Beta vulgaris* L.)

**DOI:** 10.3389/fgeed.2026.1875376

**Published:** 2026-07-21

**Authors:** Zainul A. Khan, Tinley Hathaway, Chenggen Chu, Rajeev Gupta, Melvin Bolton, Vanitharani Ramachandran

**Affiliations:** 1 The Oak Ridge Institute for Science and Education, United States Department of Agriculture, Agricultural Research Service, Fargo, ND, United States; 2 United States Department of Agriculture, Agricultural Research Service, Sugarbeet Research Unit, Edward T. Schafer Agricultural Research Center, Fargo, ND, United States; 3 Department of Plant Pathology, Microbiology and Biotechnology, North Dakota State University, Fargo, ND, United States; 4 United States Department of Agriculture, Agricultural Research Service, Cereal Crops Improvement Research Unit, Edward T. Schafer Agricultural Research Center, Fargo, ND, United States

**Keywords:** *Beta vulgaris*, gene-editing, magnesium chelatase, phytoene desaturase, sugarbeet

## Abstract

Sugarbeet (*Beta vulgaris* ssp. *vulgaris*, L.), is a vital temperate crop, supplying nearly 40% of the world’s sugar. However, its high susceptibility to bacterial, fungal, and viral diseases creates an urgent need for improved, disease-resistant cultivars. The CRISPR/Cas9 system has rapidly advanced plant genetic engineering by enabling precise and targeted genome modifications. Our goal is to establish a gene-editing platform in sugarbeet to support future development of disease-resistant lines by targeting the candidate genes. In this study, we applied CRISPR/Cas9 to generate targeted mutations in two genes involved in chlorophyll biosynthesis and carotenoid-mediated leaf pigmentation: *magnesium chelatase* (Mg-chelatase) and *phytoene desaturase* (PDS). Two CRISPR/Cas9 constructs, each carrying an sgRNA targeting either Mg-chelatase or PDS, were developed and mobilized into *Agrobacterium tumefaciens*. A total of 233 and 200 hypocotyl explants were transformed with constructs targeting Mg-chelatase and PDS, resulting in regeneration efficiencies of 8% and 14% on kanamycin selection medium, respectively. Light green, yellow, variegated yellow-green, and albino phenotypes were observed among the putative transformants, whereas non-edited transformed lines resembled untransformed control plants. Targeted mutations, including insertions, deletions, and substitutions of nucleotides, were identified at both genomic loci, with editing efficiencies of 60.0% for Mg-chelatase and 68.75% for PDS underscoring the effectiveness of this approach in sugarbeet, a recalcitrant crop. Deletions ranged from 5 to 28 bp in Mg-chelatase and 2 to 21 bp in PDS, while insertion events consisted of single-base additions in Mg-chelatase edited lines and larger insertions of 7–16 bp in PDS mutants. The results demonstrate the successful deployment of CRISPR/Cas9 for targeted genome engineering in sugarbeet and establish a reliable platform for future gene-editing efforts aimed at enhancing resistance to a wide range of pathogens and diseases affecting the crop.

## 1 Introduction

Sugarbeet (*Beta vulgaris* ssp. *vulgaris* L.), a temperate crop in the *Amaranthaceae* family (formerly *Chenopodiaceae*) contributes over 40% of global sucrose supply (Wozniak, 1999; Bekheet et al., 2007). Numerous diseases caused by viruses, fungi, and bacteria threaten sugarbeet production (Rush et al., 2006; Harveson et al., 2009; Rangel et al., 2020; Ramachandran et al., 2023a; Ramachandran et al., 2023b; Chinnadurai et al., 2024; Ramachandran et al., 2025), highlighting the need for improved varieties capable of withstanding continuously evolving pathogens. The clustered regularly interspaced short palindromic repeat (CRISPR) and CRISPR-associated 9 (Cas9) genome-editing system has revolutionized plant genome engineering by enabling efficient, targeted mutagenesis of individual genes or simultaneous editing of multiple loci (Doudna and Charpentier, 2014; Armario Najera et al., 2019; Gao, 2021). The CRISPR/Cas9 system comprises two main components: a single guide RNA (sgRNA) and the Cas9 nuclease. The sgRNA carries a 20 nucleotide (nt) sequence complementary to the target-site, which is located adjacent to the protospacer adjacent motif (PAM) recognized by Cas9 (Collias and Beisel, 2021). The *Streptococcus pyogenes* Cas9 (SpCas9) enzyme is the most frequently used endonuclease that recognize 5ʹ-NGG-3ʹ PAM sequence and introduces DNA double-strand breaks (DSBs) approximately three base pairs upstream to PAM (Doudna and Charpentier, 2014; Zhang H. X. et al., 2019). These DSBs are typically repaired through the non-homologous end joining (NHEJ) pathway, resulting in small insertions or deletions (InDels) at the repair site (Xue and Greene, 2021).

To effectively harness targeted gene-editing in sugarbeet, the establishment of CRISPR/Cas9-mediated editing requires optimizing both editing reagents and their delivery methods to achieve efficient gene targeting. This process is greatly facilitated by a rapid and easily scorable screening system that enables visual detection of targeted mutations during plant regeneration from tissue culture. In many plant species, the *phytoene desaturase* (PDS) and *magnesium chelatase* (Mg-chelatase) gene have been widely used as a visual markers for validating genome-editing platforms (Zhou et al., 2015; Zhang Y. et al., 2016; Meng et al., 2017; Jaganathan et al., 2018; Ma et al., 2019; Ntui et al., 2020; Eid et al., 2021; Nanasato et al., 2021; Ma et al., 2023). PDS is a crucial enzyme in the carotenoid biosynthesis pathway that catalyzes the desaturation of colorless phytoene into ζ-carotene, an intermediate step in the production of lycopene and other pigmented carotenoids (Bai et al., 2016). Mutations in PDS disrupt photosynthesis and the biosynthesis of gibberellins and carotenoids, resulting in easily recognizable phenotypes such as albinism and dwarfism. The Mg-chelatase catalyzes the insertion of Mg^2+^ into protoporphyrin IX, a key regulatory step in the chlorophyll biosynthesis pathway (Willows et al., 1996). In contrast to the albino and dwarf phenotypes of PDS mutants, Mg-chelatase-edited plants typically exhibit light green to yellow leaves while maintaining growth rates comparable to the wild type plants (Walker et al., 2018).

As CRISPR technologies continue to advance, the field offers precise and versatile platforms for development of targeted agronomically important traits in sugarbeet. To date, CRISPR/Cas9-mediated editing of the sugarbeet has only been demonstrated for eukaryotic translation initiation factor (iso)4E using protoplasts isolated from sugarbeet leaves to confer resistance to beet chlorosis virus (Rollwage et al., 2024). In that study, a plasmid containing the sgRNA and Cas9 was delivered into leaf-derived protoplasts. However, whole-plant regeneration from protoplasts remains a tedious and intensive process highlighting the need for a simpler and more reliable system. Here, we report the first demonstration of CRISPR/Cas9-mediated editing of the *Mg-chelatase* and *PDS* genes in sugarbeet using an *Agrobacterium*-based stable transformation approach. Cas9 nuclease and gene-specific sgRNA were delivered using a recently developed transformation and regeneration system for sugarbeet (Khan et al., 2025). Edited lines targeting Mg-chelatase exhibited yellow, light green, and variegated yellow-green phenotypes, whereas PDS-edited lines displayed albino phenotypes. Using these visually trackable marker genes allowed us to evaluate editing efficiencies, characterize the spectrum of mutagenic events, gain insight into the regeneration process leading to whole-plant recovery. Together, these findings establish a reliable genome-editing framework for targeted disruption for functional gene validation such as pathogen-responsive host genes supporting future efforts to develop enhanced pathogen resistance in sugarbeet.

## 2 Materials and methods

### 2.1 CRISPR/Cas9 vector construction

The target sites for sgRNA of *Mg-chelatase* (BI543683) and PDS (JQ085590) genes were designed using CRISPR-P 2.0 (Liu et al., 2017), CCTop - CRISPR/Cas9 target online predictor (Stemmer et al., 2015) and CRISPRdirect (Naito et al., 2015). Cas-OFFinder was used to check any potential off-target sites in the *B. vulgaris* genome (Bae et al., 2014). Mg-chelatase and PDS sgRNA target sites were ligated into pKSE401 (Addgene, MA, United States) following the Golden Gate method as described by Xing et al. (2014). To generate a sgRNA insert, 100 μmol L^-1^ each forward and reverse oligonucleotides (Supplementary Table S1) were mixed and heated at 95 °C for 5 min and cooled to 24 °C. Golden Gate reactions were set up using 200 ng pKSE401 vector, 100 ng insert, 1x T4 DNA ligase buffer, 1x BSA, 20 U *Bsa*I, 2,000 U T4 DNA ligase (New England Biolabs, MA, United States) mixed together and incubated at 37 °C for 5 h followed by 50 °C for 5 min and heat inactivation at 80 °C for 10 min. *Escherichia coli* NEB® 5-alpha (New England Biolabs, MA, United States) competent cells were transformed following manufacturer’s instructions. The recombinant clones were verified by colony PCR using U6 26p F and U6 26t R primers (Supplementary Table S1) followed by Sanger sequencing with U6 26p F primer. The resulting constructs were named pKSE401-MgCh and pKSE401-PDS for Mg-chelatase and PDS, respectively.

### 2.2 Delivery of CRISPR/Cas9 vector into *Beta vulgaris* through *agrobacterium*-mediated transformation

The pKSE401-MgCh and pKSE401-PDS plasmids were transformed into *Agrobacterium tumefaciens* GV3101 electrocompetent cells (Intact Genomics, MO, United States) following the manufacturer’s instructions, which was confirmed by colony PCR using U6 26p F and U6 26t R primers (Supplementary Table S1). A single colony carrying the individual construct was inoculated into 5 mL LB medium supplemented with 50.0 μg mL^-1^ rifampicin and 50.0 μg mL^-1^ kanamycin and incubated at 28 °C and 200 rpm for 18–24 h. Twenty µl of primary *Agrobacterium* culture was inoculated in 20 mL LB medium supplemented with antibiotics (50.0 μg mL^-1^ rifampicin and 50.0 μg mL^-1^ kanamycin) and 100 μg mL^-1^ acetosyringone, which was incubated at 28 °C with 200 rpm for 16 h. Transformation of *B. vulgaris* (KWSH_01; kindly provided by KWS, Einbeck, Germany) was performed using hypocotyl explants of 10 day old sugarbeet seedlings grown on Murashige and Skoog (MS) medium following the methods described previously (Khan et al., 2025). All transformed explants were transferred to callus induction medium (CIM), which consisted of MS medium pH 5.8 with 3% sucrose supplemented with 2.0 mg l^-1^ N6-benzylaminopurine (BAP), 250 mg L^-1^ cefotaxime, 300 mg L^-1^ timentin, 50 mg L^-1^ kanamycin, 1.0 μL mL^-1^ Plant Preservative Mixture (PPM; Plant Cell Technology, Washington DC, United States), and solidified with 0.8% agar. After three to 4 weeks, the kanamycin resistant calli were transferred to shoot induction medium (SIM) containing 1.0 mg L^-1^ BAP, 1.0 mg L^-1^ kinetin, 0.1 mg L^-1^ α-naphthaleneacetic acid (NAA), 250 mg L^-1^ cefotaxime, 300 mg L^-1^ timentin, 50 mg L^-1^ kanamycin, and 1.0 μL mL^-1^ PPM. The regenerated shoots were subsequently transferred to root induction medium (RIM) supplemented with 3.0 mg L^-1^ NAA, 3.0 mg L^-1^ IAA and 1.0 μL mL^-1^ PPM. The regenerated sugarbeet plantlets were visually assessed for phenotypes resulting from the knockout of *Mg-chelatase* and *PDS* gene functions.

### 2.3 Molecular confirmation of *Beta vulgaris* transgenic lines

Total genomic DNA was extracted from transformed and non-transformed sugarbeet plants, using the DNeasy® Plant Mini Kit following the manufacturer’s instructions (Qiagen, MD, United States). DNA quality and quantity were assessed by using a Nanodrop 2000 Spectrophotometer (Thermo Scientific, MA, United States). PCR was carried out in a 20 µL reaction volume containing 1X Phusion HF Buffer, 200 µM dNTPs, 0.5 µM each of forward and reverse primers (Supplementary Table S1), 100 ng of genomic DNA and 0.5 U of Phusion DNA Polymerase (New England Biolabs, MA, United States). PCR conditions to amplify *neomycin phosphotransferase II* (*NPT II*) gene include an initial denaturation at 98 °C for 30 s, followed by 30 cycles of denaturation at 98 °C for 10 s, annealing at 59 °C for 30 s, and extension at 72 °C 30 s, with a final extension at 72 °C for 10 min. PCR amplicons were resolved on 1.2% (w/v) agarose gel stained with Safe DNA Gel Stain (APExBIO, TX, United States) and visualized using the ChemiDocTM MP Imaging System (Bio-Rad Laboratories, CA, United States). The expected DNA bands were excised from the gel and DNA was eluted using the QIAquick® Gel Extraction Kit (Qiagen, MD, United States) according to the manufacturer’s instructions. The gel purified DNA was then subjected to Sanger sequencing using gene specific primers to obtain the sequence information (Eurofins Genomics, KY, United States).

### 2.4 Sequence analysis of targeted mutations in *Mg-chelatase* and *PDS* genes

The target sequences for Mg-chelatase and PDS were amplified from transformed and non-transformed sugarbeet plants using specific primers mentioned in Supplementary Table S1. PCR was carried out in a 20 µL reaction volume containing 1X Phusion HF Buffer, 200 µM dNTPs, 0.5 µM each of forward and reverse primers (Supplementary Table S1), 100 ng of genomic DNA and 0.5 U of Phusion DNA Polymerase (New England Biolabs, MA, United States). PCR conditions for amplifying the *Mg-chelatase* and *PDS* genes included an initial denaturation at 98 °C for 30 s followed by 30 cycles of denaturation at 98 °C for 10 s, annealing at 52.6 °C for Mg-chelatase and 50.0 °C for PDS for 30 s, and extension at 72 °C 30 s, with a final extension at 72 °C for 10 min. PCR amplicons were resolved on a 1.3% (w/v) agarose gel stained with Safe DNA Gel Stain (APExBIO, TX, United States) and visualized using the ChemiDoc™ MP Imaging System (Bio-Rad Laboratories, CA, United States). The expected DNA bands were excised from the gel, and DNA was purified using the QIAquick® Gel Extraction Kit (Qiagen, MD, United States) according to the manufacturer’s instructions. PCR purified target sequences of Mg-chelatase and PDS were sequenced using Sanger technology (Eurofins Genomics, KY, United States). Mutations in the sugarbeet target sequences were analyzed from the Sanger chromatograms using the online ICE CRISPR Analysis Tool (EditCo) as previously described by (Gong et al., 2025). In addition, the EditR tool was used to detect base editing events from the Sanger chromatograms of the target sequences (Kluesner et al., 2018). The gene-editing efficiency was calculated using NPT II-confirmed lines.

## 3 Results

### 3.1 Identification of target sites and construction of editing vectors

**FIGURE 1:**
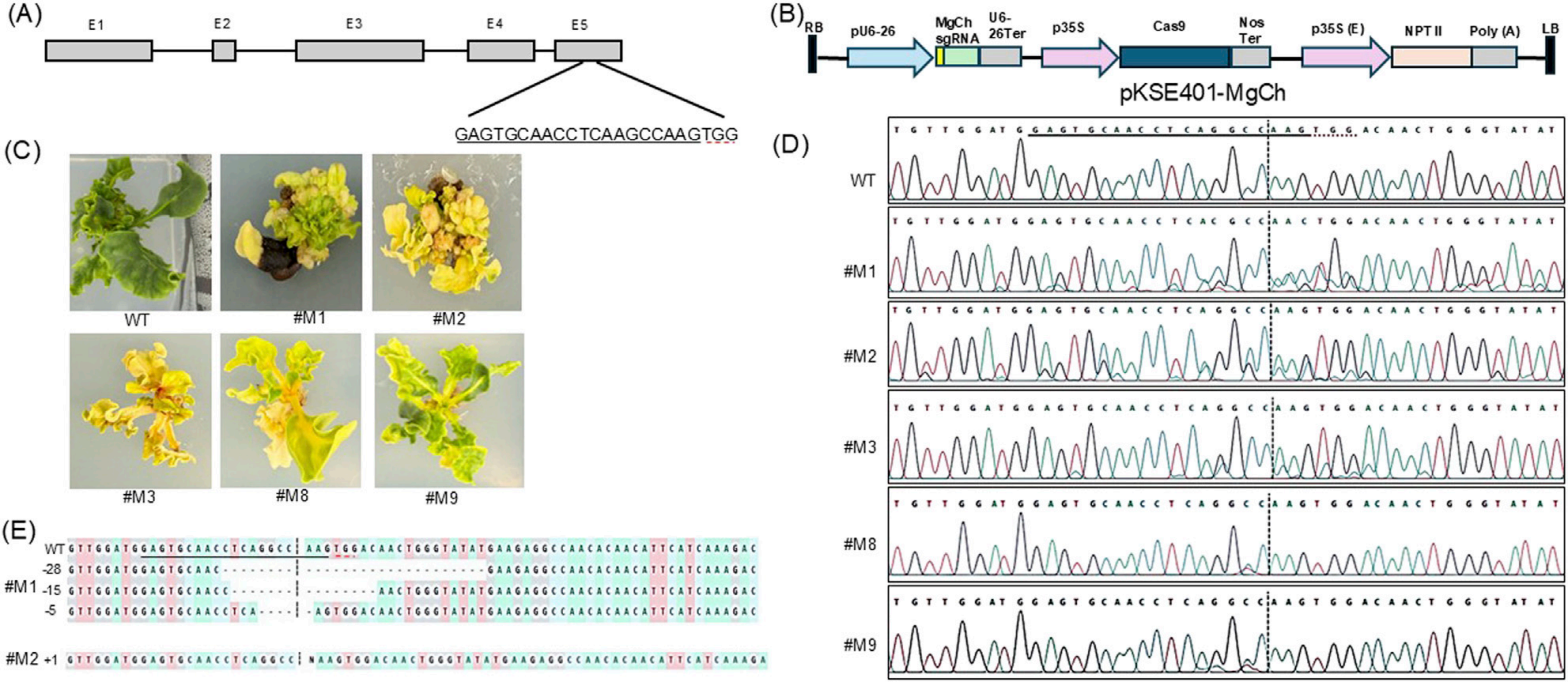
CRISPR/Ca9-mediated editing of the *Mg-chelatase* gene in *Beta vulgaris* **(A)** Gene structure of Mg-chelatase of *B. vulgaris*, showing the sgRNA target-site. Grey blocks indicate exons and lines represent introns of the gene **(B)** Schematic representation of CRISPR/Cas9 binary vector construct having Cas9 and sgRNA cassettes for Mg-chelatase along with NPT II **(C)** CRISPR/Cas9-directed alteration of *Mg-chelatase* gene function on phenotypes of regenerated sugarbeet plants. Yellow, green and variegated yellow-green phenotypes appear in Mg-chelatase-edited sugarbeet lines compared to green wild-type (WT) control **(D)** Sanger sequencing chromatograms of the target region of Mg-chelatase edited and WT sugarbeet lines **(E)** Mutations at Mg-chelatase target sites show different length of deletions. The sgRNA sequence for Mg-chelatase is underlined with black solid line and the PAM is underlined with dotted red line. Vertical dotted lines represent predicted cleavage site 3 bp upstream of PAM.

**FIGURE 2:**
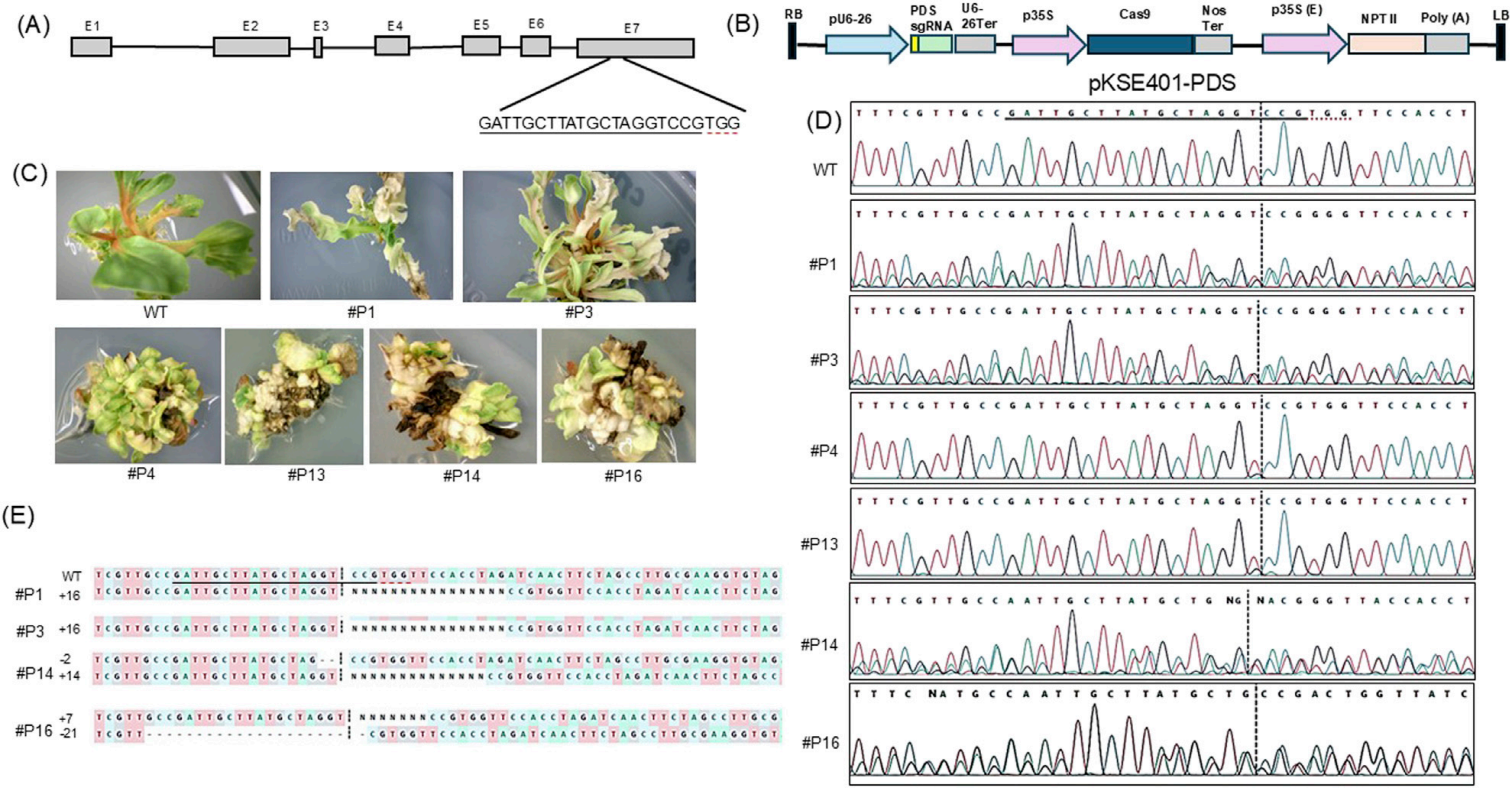
CRISPR/Ca9-mediated editing of the *PDS* gene in *Beta vulgaris* **(A)** Gene structure of PDS of *B. vulgaris*, showing the sgRNA target. Grey blocks indicate exons and lines represent introns of the gene **(B)** Schematic representation of CRISPR/Cas9 binary vector construct containing Cas9 and sgRNA for PDS along with NPT II **(C)** CRISPR/Cas9-directed alteration of *PDS* gene function on phenotypes of regenerated sugarbeet plants. Albino and green-white phenotypes display in PDS-edited sugarbeet lines compared to green wild-type (WT) control **(D)** Sanger sequencing chromatograms of the target region of PDS edited and WT sugarbeet lines **(E)** Mutations at PDS target sites show different lengths of insertions and deletions. The sgRNA sequence for PDS target is underlined with black solid line and the PAM is underlined with dotted red line. Vertical dotted lines represent predicted cleavage site 3 bp upstream of PAM.

The complete genomic sequences of the *Mg-chelatase* (NC_079204) and *PDS* (NC_079205) genes span 6,325 and 4,299 bp and are located on chromosomes 3 and 4, respectively. Gene structure analysis revealed that *Mg-chelatase* comprises five exons and four introns, whereas the *PDS* gene contains seven exons and six introns (Figures 1A, 2A). Partial gene sequences previously deposited in GenBank for *Mg-chelatase* (BI543683.1) and *PDS* (JQ085590) of *B. vulgaris* were used as references during initial target identification. Two sgRNAs were designed to target exon 5 of *Mg-chelatase* and exon 7 of *PDS*, respectively (Figures 1A, 2A). To evaluate target specificity and eliminate potential off-target effects, both sgRNAs were analyzed using BLAST and Cas-OFFinder against *B. vulgaris* subsp. *vulgaris* genome (taxid:3,555). No predicted off-target sites were detected for either sgRNA, indicating high target specificity and suitability for CRISPR/Cas9-mediated editing of Mg-chelatase and PDS in the *B. vulgaris* genome. As no off-target sites were predicted in the *in silico* analysis, experimental evaluation of potential off-target effects was not pursued. Each sgRNA was subsequently cloned into the pKSE401 binary vector and sequencing of the recombinant constructs confirmed correct insertion of the sgRNA expression cassette within the T-DNA region. The complete sgRNA cassettes, including the U6 promoter, sgRNA spacer and scaffold, and U6 terminator sequences are shown in Supplementary Figures S1, S2. The final constructs, each containing the Cas9, sgRNA, and NPT II expression cassettes targeting Mg-chelatase or PDS, are designated pKSE401-MgCh and pKSE401-PDS, respectively (Figures 1B, 2B).

### 3.2 Generation of transgenic *Beta vulgaris* lines containing CRISPR/Cas9 and phenotype analysis of edited plants

**FIGURE 3:**
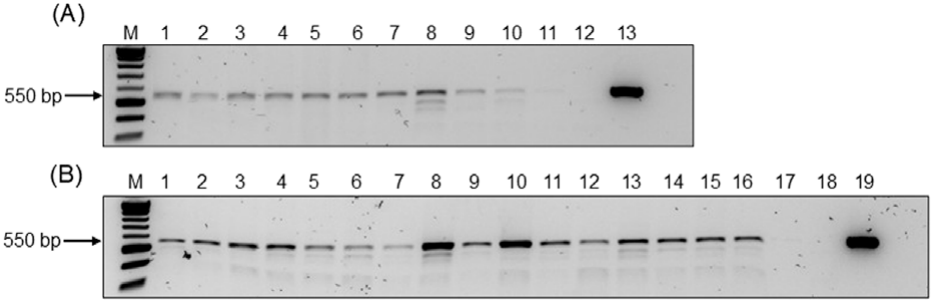
PCR-based detection of the *neomycin phosphotransferase II* (*NPT II*) gene transformed *Beta vulgaris* lines **(A)** Transgene confirmation by PCR amplification of NPT II using specific primers produced a 550 bp DNA fragment in Mg-chelatase transformed lines (lane 1–10). Lane 11: non-transformed sugarbeet; lane 12: non-template negative control; lane 13: positive control (pKSE401-MgCh plasmid) **(B)** Transgene confirmation by PCR amplification of NPT II using specific primers produced a 550 bp DNA fragment in PDS transformed lines (lane 1–16). Lane 17: non-transformed sugarbeet; lane 18: non-template negative control; lane 19: positive control (pKSE401-PDS plasmid). Lane M: 1 kb plus DNA ladder.

An amplicon of approximately 423 bp obtained by colony PCR using U6 26p F and U6 26t R primers confirmed the successful mobilization of the pKSE401-MgCh and pKSE401-PDS constructs into *A. tumefaciens* GV3101. For the sugarbeet transformation, the protocol including explant type and age, *Agrobacterium* culture optical density, infection duration, and cocultivation period were followed as described previously (Khan et al., 2025). Transformation was performed using 233 hypocotyl explants for pKSE401-MgCh and 200 explants for pKSE401-PDS (Table 1; Supplementary Figure S3). After 3–4 weeks on CIM containing 50 mg L^-1^ kanamycin, 19.3% of explants transformed with pKSE401-MgCh and 32.5% of explants transformed with pKSE401-PDS survived on selection medium (Table 1; Supplementary Figure S3). Following an additional three to 4 weeks on SIM with the same kanamycin concentration, the regeneration rates were 42.2% for pKSE401-MgCh-transformed explants and 43.1% for pKSE401-PDS-transformed explants (Table 1; Supplementary Figure S3). Integration of the NPT II selectable marker in the regenerated lines was confirmed by a 550 bp PCR amplicon and Sanger sequencing (Figures 3A,B).

**TABLE 1:** Transformation and editing efficiencies and associated phenotypes in *Mg-chelatase* and *PDS* genes-edited sugarbeet lines using CRISPR/Cas9.

Construct	Number of explants transformed	Number of explants resistant to kanamycin	Number of regenerated plants	Phenotypes of regenerated sugarbeet lines					Number of plants with NPT II gene	Number of NPT II positive plants showing editing events	Genome editing efficiency (%)	Number of edited plants showing altered pigmentation phenotypes^2^
				Yellow	Green	Variegated yellow green	White	Variegated green white				
pKSE401-MgCh	233	45	19	4	9	6	ND^1^	ND	10	6	60	5
pKSE401-PDS	200	65	28	4	5	7	5	7	16	11	68.75	6

1 ND: not detected.

2 The edited plants that display altered pigmentation phenotypes are presented in Figures 1C, 2C.

Plantlets regenerated from explants transformed with pKSE401-MgCh exhibited visually distinct chlorophyll depletion immediately upon regeneration. These events were characterized by light green or yellow leaves, in contrast to the dark green leaves of non-transformed control plants and NPT II-positive, non-edited plants (Figure 1C). Among the Mg-chelatase-targeted events, four plantlets exhibited a fully yellow phenotype, six showed a mixture of yellow and light green leaves, and nine remained green (Table 1). Similarly, regeneration of pKSE401-PDS-transformed explants resulted in diverse pigmentation phenotypes: five events were fully albino, seven showed a mixture of green and white, four events were yellow, seven displayed yellow and green variegation, and five remained green (Figure 2C; Table 1).

The regenerated sugarbeet plantlets were transferred to RIM for further development. The yellow plants as well as those showing a mixture of yellow and green pigmentation grew slowly and started producing roots after 19 weeks on RIM after several subcultures, regardless of whether they were transformed with pKSE401-MgCh or pKSE401-PDS. In contrast, green or light-green pKSE401-MgCh transformants initiated roots after about 10 weeks on RIM. Non-edited sugarbeet lines rooted more efficiently, with root induction occurring within two to three weeks on RIM. A clear phenotypic difference between Mg-chelatase-edited and non-edited lines was observed after 17 weeks of subculture on RIM. The delayed root formation observed in the Mg-chelatase- and PDS-edited lines may result from disruptions of chlorophyll-biosynthesis genes, which may be involved in regulating root development. The Mg-chelatase-edited line exhibited a yellow phenotype with small leaves and stunted growth, whereas the non-edited line displayed a green phenotype and grew normally, like wild-type sugarbeet plants. Similarly, PDS-edited lines displayed albino phenotypes, clearly distinguishing them from non-edited transformed lines and these albino plants showed delayed root formation.

### 3.3 Detection of CRISPR-induced mutations in edited plants by sanger sequencing

Ten NPT II PCR positive events of pKSE401-MgCh and 16 events of pKSE401-PDS were selected for analysis to detect mutations in target region by Sanger sequencing. PCR amplification of the target regions generated 250 bp products for Mg-chelatase and 470 bp products for PDS, and sequencing confirmed successful amplification both in putative transgenic and non-transformed sugarbeet plants (Supplementary Figure S4). Sanger sequencing chromatograms of these amplicons revealed overlapping peaks at the expected CRISPR cleavage sites for both Mg-chelatase and PDS, indicating the presence of mutations at these target sites (Figures 1D, 2D).

In the Mg-chelatase mutant line #M1, deletions of 5, 15 and 28 nucleotides were detected, whereas the #M2 mutant line exhibited a single-nucleotide insertion located 3 bp upstream of the PAM (Figure 1E; Supplementary Table S2). In mutant line #M2, overlapping chromatogram peaks at positions 1, 3, 5, 8, and 15 bp upstream of the PAM site indicate base substitutions of G→C, A→C, C→G, A→C, and C→G, respectively. The predominant nucleotide frequencies at these positions were 57% (G), 65% (A), 65% (C), 55% (A), and 65% (C) (Supplementary Figure S5; Supplementary Table S2). In the #M3 mutant line, overlapping chromatogram peaks at positions 3 bp and 7 bp upstream of the PAM indicated base substitutions (A→C and G→C), with dominant nucleotide frequencies of 70% (A) and 60% (G), respectively. Similarly, in mutant line #M9 showed overlapping peaks at 5 bp and 7 bp upstream of the PAM base substitutions C→T and G→C, with dominant nucleotide frequencies of 62% (C) and 76% (G), respectively. A single overlapping peak at 5 bp upstream of the PAM was detected in mutant lines #M8, indicating a C→T substitution, with dominant C frequency of 67% (Figure 1D; Supplementary Figure S5; Supplementary Table S2).

Insertion-deletion (InDel) mutations were detected in PDS mutant lines #P14 and #P16. Line #P14 carried a 14 bp insertion and a 2 bp deletion located 3 bp upstream of the PAM. Similarly, line #P16 contained a 7 bp insertion along with a 21 bp deletion, with the deletion beginning 2 bp upstream of the PAM (Figures 2D,E; Supplementary Table S2). A 16 bp insertion was also observed in mutant lines #P1 and #P3 (Figure 2E). In mutant line #P13, overlapping chromatogram peaks at positions 3 bp and 4 bp upstream of the PAM indicated base substitutions (C→A and T→G), with dominant nucleotide frequencies of 83% (C) and 73% (T), respectively. A single overlapping peak at 4 bp upstream of the PAM was observed in mutant line #P4, corresponding to a T→G substitution with a dominant T frequency of 79% (Figure 2D; Supplementary Figure S6; Supplementary Table S2). Additionally, a T→G substitution at the first PAM nucleotide was detected in mutant lines #P1, #P3 and #P14 (Figure 2D).

## 4 Discussion

The CRISPR/Cas9 system has proven to be a versatile tool for functional genomics research, enabling the precise development of agriculturally important traits across diverse crops. Genome-editing offers significant potential for developing improved sugarbeet varieties capable of resisting diverse pathogens, including viruses, and pests (Rush et al., 2006; Harveson et al., 2009; Rangel et al., 2020; Ramachandran et al., 2023a; 2023b; Chinnadurai et al., 2024; Ramachandran et al., 2025). In this study, we developed a CRISPR/Cas9-mediated gene-editing system development for sugarbeet, targeting two host reporter genes, *Mg-chelatase* and *PDS*. Disruption of these genes interferes with key steps in chlorophyll and carotenoid biosynthesis, resulting in yellow or albino phenotypes that provide an immediate, non-destructive readout of editing efficiency (Qin et al., 2007; Walker et al., 2018). The reproducibility and clarity of these phenotypes have made Mg-chelatase and PDS visual markers for assessing genome-editing across numerous plant species (Zhou et al., 2015; Zhang Y. et al., 2016; Meng et al., 2017; Jaganathan et al., 2018; Ma et al., 2019; Ntui et al., 2020; Eid et al., 2021; Nanasato et al., 2021; Ma et al., 2023). Therefore, demonstrating their utility in sugarbeet is a critical step toward establishing a reliable and generalized editing framework for the crop. To build such a framework, we developed CRISPR/Cas9 reagents, including sgRNAs targeting Mg-chelatase and PDS and Cas9 enzyme-containing constructs. BLAST analysis confirmed both genes exist as single-copy loci in the *B*. *vulgaris* genome, which is crucial for achieving on-target mutagenesis without confounding effects from functional redundancy or gene family compensation. These constructs were transformed into hypocotyl explants via *Agrobacterium*-mediated delivery as described previously (Khan et al., 2025). The single-copy architecture of PDS is consistent with findings in multiple crop species such as cassava (Odipio et al., 2017), cavendish banana (Naim et al., 2018), melon (Hooghvorst et al., 2019) and onion (Mainkar et al., 2023).

The development of a CRISPR/Cas9 editing system offers crucial biological and applied insights for building a consistent gene-editing platform in sugarbeet. However, establishing this platform presents significant challenges. Genetic transformation in sugarbeet, despite its critical role in crop improvement, is notoriously difficult, primarily due to the crop’s inherent recalcitrance in tissue culture and regeneration, coupled with genomic complexity (Snyder et al., 1999; Zhang C. L. et al., 2001; Ivic-Haymes and Smigocki, 2005; Tehseen et al., 2024). Over the years, multiple transformation approaches that includes different delivery methods followed by direct-shoot regeneration resulted in varying degrees of success in sugarbeet (Lindsey and Jones, 1989; Hall et al., 1996; Hall et al., 1997; Gurel et al., 2002; Hisano et al., 2004; Yang et al., 2005; De Marchis et al., 2009; Jafari et al., 2009; De Marchis et al., 2009; Moazami-Goodarzi et al., 2020; Zhang Z. et al., 2023). Although protoplast-based endogenous gene modification has been shown in sugarbeet and other crops, this approach remains unreliable for routine or large-scale applications particularly recalcitrant plant like sugarbeet (Li et al., 2021; Lin et al., 2022; Rollwage et al., 2024; Tricoli and Debernardi, 2023; Barrera et al., 2025). Protoplasts are difficult to obtain in consistent quantities, highly sensitive once isolated, and exhibit challenges including low recovery of fully regenerated edited plants. These limitations highlight the need for a more dependable transformation and regeneration system to support reliable gene-editing system development in sugarbeet. *Agrobacterium*-mediated transformation remains an economical and widely accessible approach, and callus-based regeneration offers the advantage of generating multiple, independent transformation events from a relatively homogenous tissue source. In this context, the hypocotyl-derived callus system recently developed in our laboratory (Khan et al., 2025) offers a more consistent and efficient, and uniform platform for sugarbeet transformation. This dependable source of transformable tissue is crucial for enabling the exploration of CRISPR/Cas9-mediated genome-editing within the same cultivar. This consistency can accelerate the development of precise, genotype-specific gene-editing strategies in sugarbeet.

Using this system, we successfully regenerated 19 Mg-chelatase and 28 PDS transformed sugarbeet lines with varying levels of phenotypes. Mg-chelatase-edited plants carried deletions or insertions, indicated by multiple overlapping peaks in the target regions, and exhibited pronounced yellow phenotypes. In contrast, edited plants with a single overlapping peak 5 bp upstream of the PAM displayed light green or variegated yellow-green phenotypes. Although the Mg-chelatase reference sequence used for sgRNA design (BI543683.1) contains an adenine at the seventh position upstream of the PAM, Sanger sequencing of sugarbeet material showed this position is guanine. The difference likely reflects variation among sequence sources rather than polymorphism. Notably, sgRNA with a mismatch at this position is reported to have only minimal effect on editing efficiency (Zheng et al., 2017). As with Mg-chelatase-edited plants, PDS-edited lines with multiple overlapping peaks in the target region showed albino phenotypes, while those edited plants with a single overlapping peak showed green-and-white variegation. These phenotypic patterns are consistent with CRISPR/Cas9-induced disruptions in the *PDS* gene in banana and poplar (Naim et al., 2018; Wang et al., 2020) and with Mg-chelatase knockout studies reported across diverse plant species (Eid et al., 2021; Nanasato et al., 2021; Ma et al., 2023). Together, these results support the reliability of our transformation and editing platform for generating stable, edited sugarbeet lines.

Although InDels generated through the NHEJ pathway are typically considered the predominant mutation type in CRISPR/Cas9-mediated gene-editing, our results also revealed the presence of single-nucleotide substitutions within the target regions of both *Mg-chelatase* and *PDS*. The occurrence of high substitution mutation rates following CRISPR/Cas9-editing is well documented across several crop species. Significant levels have been documented in *Glycine* *max* and *Gossypium hirsutum* (Sun et al., 2015; Chen et al., 2017), and in some cases, such as cassava, substitutions were even more prevalent than InDels (Odipio et al., 2017). Further examples include melon (91% substitutions; Hooghvorst et al., 2019), *Oryza sativa* L (25%–45%; Macovei et al., 2018), and onion (55%; Mainkar et al., 2023). Collectively, these observations suggest that substitution events may be a more common outcome of CRISPR/Cas9-mediated mutagenesis in plants than previously recognized, potentially influenced by species-specific DNA repair landscapes, target sequence context, or chromatin architecture. While Sanger sequencing remains a widely used and accessible approach for characterizing CRISPR-induced variants, mixed chromatogram peaks from heterozygous or chimeric tissues can compromise accuracy and hinder the detection of low-frequency edits, thereby reducing sensitivity in complex lines.

The InDels identified in the *Mg-chelatase* and *PDS* target regions in this study align with NHEJ-driven repair of CRISPR/Cas9-induced double-strand DNA breaks, consistent with numerous studies targeting these genes across diverse plant species (Fan et al., 2015; Zhou et al., 2015; Nishitani et al., 2016; Zhang Y. et al., 2016; Meng et al., 2017; Jaganathan et al., 2018; Ma et al., 2019; Ntui et al., 2020; Eid et al., 2021; Nanasato et al., 2021; Ma et al., 2023). Simultaneous activation of both NHEJ and HR pathways has been previously reported in apple, cotton and *Nicotiana benthamiana* (Li et al., 2013; Nishitani et al., 2016; Gao et al., 2017). Collectively, these findings support the interpretation that sugarbeet employs multiple endogenous DNA-repair mechanisms in response to CRISPR-mediated editing, contributing to a broader spectrum of mutation outcomes. In the present study, primary transformants were analyzed, which may include both somatic and heritable editing events. *Agrobacterium*-mediated transformation of hypocotyl explants produces callus derived from numerous dividing cells, resulting in T0 plants that are mosaics containing wild-type and variously edited cell lineages (Jang et al., 2016; Lee et al., 2019). Since mutation analysis in this work is limited to T0 plants, evaluation of T1 and T2 generations will be necessary to determine the extent of chimera resolution and genotype segregation. A practical constraint is that sugarbeet is a biennial crop; thus, completing the generational assessments requires two growing seasons to accommodate both vegetative and reproductive phases needed for heritability analyses.

The gene-editing system developed here, targeting *Mg-chelatase* and *PDS*, establishes a reliable platform that can be readily extended for functional genomics studies and the development of novel agronomic traits in sugarbeet. By enabling efficient and reproducible editing in sugarbeet, this system provides a powerful tool for generating improved pre-breeding germplasm and accelerating cultivar development. This platform lays essential groundwork for traits such as enhanced pathogen resistance including metabolic modifications, thereby supporting long-term efforts to advance sugarbeet improvement through precision genome engineering.

## Data Availability

The original contributions presented in the study are included in the article/Supplementary Material, further inquiries can be directed to the corresponding author.
