# Supplementary material for: CRISPR/Cas9-based gene-editing platform development by targeting genes encoding magnesium chelatase and phytoene desaturase in sugarbeet (Beta vulgaris L.)
Source: Front Genome Ed. 2026 Jul 21;8:1875376. doi: 10.3389/fgeed.2026.1875376 (PMC13433599; doi:10.3389/fgeed.2026.1875376)
Supplement: Supplementary file 1 [file Supplementaryfile1.docx]

Supplementary Material

# 1 Supplementary Figures and Tables

Supplementary Table S1 List of primers used for sgRNA cloning and PCR

| Name | Sequences (5ˊ→3´) | Description | Reference |
| --- | --- | --- | --- |
| MgCh For | ATTGGAGTGCAACCTCAAGCCAAG | sgRNA cloning | This study |
| MgCh Rev | AAACCTTGGCTTGAGGTTGCACTC | SgRNA cloning |  |
| MgCh Tar For | ACGAGGCAACGCACAGGTAC | MgCh target amplification |  |
| MgCh Tar Rev | ACCCCTTCCATTGGCCTCTA | MgCh target amplification |  |
| PDS For | ATTGGATTGCTTATGCTAGGTCCG | SgRNA cloning |  |
| PDS Rev | AAACCGGACCTAGCATAAGCAATC | SgRNA cloning |  |
| PDS Tar For | GCGTGCCAGGATGTTCTTTG | PDS target amplification |  |
| PDS Tar Rev | ACACAGCTCATGCTTTACAATG | PDS target amplification |  |
| NPT II For2 | TTGGGTGGAGAGGCTATTCGGCTAT | Transgene amplification |  |
| NPT II Rev2 | ATGATATTCGGCAAGCAGGCATCGC | Transgene amplification |  |
| U6 26p F | TGTCCCAGGATTAGAATGATTAGGC | Colony PCR | Xing et al. 2014 |
| U6 26t R | CCCCAGAAATTGAACGCCGAAGAAC | Colony PCR |  |
| U6 26p F | TGTCCCAGGATTAGAATGATTAGGC | Sequencing |  |

Supplementary Table S2 Summary of mutation characteristics detected in magnesium-chelatase edited *Beta vulgaris* lines

| Edited lines | Mutation type | Size of InDel | Position (start - end) | Nucleotide (%) in substitution mutation |
| --- | --- | --- | --- | --- |
| M1 | Deletion | -28 | 11 bp upstream - 14 bp downstream of PAM | NA |
|  | Deletion | -15 | 10 bp upstream - 2 bp downstream of PAM | NA |
|  | Deletion | -5 | 3 bp - 7 bp upstream of PAM | NA |
| M2 | Insertion | +1 | 3 bp upstream of PAM | NA |
|  | Substitution (G→C) |  | 1 bp upstream of PAM | G (57) |
|  |  |  |  | C (43) |
|  | Substitution (A→C) |  | 3 bp upstream of PAM | A (65) |
|  |  |  |  | C (35) |
|  | Substitution (C→G) |  | 5 bp upstream of PAM | C (65) |
|  |  |  |  | G (35) |
|  | Substitution (A→C) |  | 8 bp upstream of PAM | A (55) |
|  |  |  |  | C (45) |
|  | Substitution (C→G) |  | 15 bp upstream of PAM | C (65) |
|  |  |  |  | G (35) |
| M3 | Substitution (A→C) |  | 3 bp upstream of PAM | A (70) |
|  |  |  |  | C (30) |
|  | Substitution (G→C) |  | 7 bp upstream of PAM | G (60) |
|  |  |  |  | C (40) |
| M4 | Substitution (C→T) |  | 5 bp upstream of PAM | C (62) |
|  |  |  |  | T (38) |
| M8 | Substitution (C→T) |  | 5 bp upstream of PAM | C (67) |
|  |  |  |  | T (33) |
| M9 | Substitution (C→T) |  | 5 bp upstream of PAM | C (62) |
|  |  |  |  | T (38) |
|  | Substitution (G→C) |  | 7 bp upstream of PAM | G (76) |
|  |  |  |  | C (24) |
| P1 | Insertion | +16 | 3 bp upstream of PAM | NA |
|  | Substitution (T→G) |  | 1^st^ PAM nucleotide | NA |
| P3 | Insertion | +16 | 3 bp upstream of PAM | NA |
|  | Substitution (T→G) |  | 1^st^ PAM nucleotide | NA |
| P4 | Substitution (T→G) |  | 4 bp upstream of PAM | T (79) |
|  |  |  |  | G (21) |
| P13 | Substitution (C→A) |  | 3 bp upstream of PAM | C (83) |
|  |  |  |  | A (17) |
|  | Substitution (T→G) |  | 3 bp upstream of PAM | T (73) |
|  |  |  |  | G (27) |
| P14 | Insertion | +14 | 3 bp upstream of PAM | NA |
|  | Deletion | -2 | 4 bp upstream - 5 bp upstream of PAM | NA |
|  | Substitution (T→G) |  | 1^st^ PAM nucleotide | NA |
| P16 | Insertion | +7 | 3 bp upstream of PAM | NA |
|  | Deletion | -21 | 2 bp upstream - 23 bp upstream of PAM | NA |

NA: Not applicable

M: Magnesium-chelatase lines

P: PDS lines

## 1.1 Supplementary Figures
